# Supplementary material for: Modelling the linkage between coral assemblage structure and pattern of environmental forcing
Source: R Soc Open Sci. 2020 Oct 14;7(10):200565. doi: 10.1098/rsos.200565 (PMC7657928; doi:10.1098/rsos.200565)
Supplement: Appendix S1 [file rsos200565supp1.pdf]

Appendix S1

A multivariate regression tree is constructed by iteratively splitting the classes in order to maximize the decrease of the deviance. In this work, the deviance formulation for each class *p* of the tree is:

$$\hat{R}(p) = \sum_{X_k \in p} \left\| X_k - \bar{X}_p \right\|^2$$

where $\bar{X}_p$ is the mean of the observations of *X* belonging to region *p*, with $\|.\|$ the usual norm in $\mathbb{R}^n$. Starting with the whole sample of the response variable space, where *j* is a counter of across the set of all model realizations, let us consider a splitting variable ψ_j and a threshold *s* on this variable. We then define the region p_1 for which $\psi_j \leq s,\ p_1 = \{X_{k=1,\dots,r} | \psi_j \leq s\}$, and the region p_2 for which $\psi_j > s,\ p_2 = \{X_{k=1,\dots,r} | \psi_j > s\}$, such that $p = p_1 \cup p_2$ and $p_1 \cap p_2 = \varnothing$. The within class sum of squares can be calculated in each of these parts of m-orthotopes:

$$\hat{R}(p_1) = \sum_{X_k \in p_1} \left\| X_k - \bar{X}_{p_1} \right\|^2$$

and

$$\hat{R}(p_2) = \sum_{X_k \in p_2} \left\| X_k - \bar{X}_{p_2} \right\|^2$$

For any split s belonging to the set S of all candidate splits, p is subdivided into p_1 and p_2, and the variation in deviance is given by

$$\Delta\hat{R}(s,p)=\hat{R}(p)-\left[\hat{R}(p_1)+\hat{R}(p_2)\right].$$

The selected split s^* of p into p_1 and p_2 is the split that most decreases $\hat{R}(p_1)+\hat{R}(p_2)$ such that

$$\Delta\hat{R}(s^*,p)=\max_{s\in S}\Delta\hat{R}(s,p)$$

The decreases in $\hat{R}(p)$ when splitting a region p into p_1 and p_2 is guaranteed because the following property is verified for any p:

$$\hat{R}(p)=\hat{R}(p_1)+\hat{R}(p_2)+\frac{r_1r_2}{r_1+r_2}\sum_{X_k\in p_1}\left\|\bar{X}_{p_1}-\bar{X}_{p_2}\right\|^2$$

with r_1 and r_2 the number of observations respectively in p_1 and p_2. This property arising from the decomposition of the inertia and the Huygens theorem is verified because the criterion $\hat{R}(p)$ is a sum of squared distances. The tree is grown until the decrease in deviance is inferior or equal to 2%.
